# Supplementary material for: Putting computational models of immunity to the test—An invited challenge to predict B.pertussis vaccination responses
Source: PLoS Comput Biol. 2025 Mar 31;21(3):e1012927. doi: 10.1371/journal.pcbi.1012927 (PMC11978014; doi:10.1371/journal.pcbi.1012927)
Supplement: S1 Text — Table A. Antibody information. Table B. The characteristics of all 21 subjects in the challenge dataset. Fig A: Plot of assay data before and after normalization and batch effect correction. For each assay, the plots on the left represent data before batch correction, while the plots on the right represent data after normalization and batch correction. Fig B: Schematic representation of control model construction. (a) Control model using subject age: Raw age values (orange) are ranked (blue) to create a ranked list based on age. (b) The control model uses IgG-PT levels on Day 0 for Task 1.1: Raw IgG-PT values (red) are ranked (blue) to represent the relative position of subjects based on their pre-vaccination IgG-PT levels. Similarly, monocyte frequency on Day 1 was used to construct the control model for Task 2.1, and CCL3 levels on Day 3 were used to construct the control model for Task 3.1. (c) Control model using the inverse of IgG-PT levels on Day 0 for Task 1.2: Raw IgG-PT values (red) are converted into negative values, then ranked (blue), emphasizing individuals with lower IgG-PT levels. Similarly, the negative values of monocyte frequency on Day 1 were used to construct the control model for Task 2.2, and the negative values of CCL3 levels on Day 3 were used to construct the control model for Task 3.2. Fig C: The method implemented by the Dr. Thrupp’s team (user48_1). (a) The analysis pipeline begins with batch-corrected data for both training and prediction phases. The training data includes all four assay data provided, i.e., Plasma Antibody levels, PBMC Gene expression, PBMC Cell frequency, and Plasma Cytokine concentrations. Similarly, prediction data is derived from the same types of measurements. b) Multi-omics factor Analysis (MOFA) is employed for multi-omics data integration through dimensionality reduction. The input data (X1, X2, X3, X4) represent different omics datasets (e.g., gene expression, cell frequency, cytokine levels, and antibody level [file pcbi.1012927.s001.pdf]

Supplemental information

Putting computational models of immunity to the test - an invited challenge to predict *B. pertussis* vaccination outcomes

Pramod Shinde, Lisa Willemsen, Michael Anderson, Minori Aoki, Saonli Basu, Julie G Burel, Peng Cheng, Souradipto Ghosh Dastidar, Aidan Dunleavy, Tal Einav, Jamie Forschmiedt, Slim Fourati, Javier Garcia, William Gibson, Jason A Greenbaum, Leying Guan, Weikang Guan, Jeremy P Gygi, Brendan Ha, Joe Hou, Jason Hsiao, Yunda Huang, Rick Jansen, Bhargob Kakoty, Zhiyu Kang, James J Kobie, Mari Kojima, Anna Konstorum, Jiyeun Lee, Sloan A Lewis, Aixin Li, Eric F Lock, Jarjapu Mahita, Marcus Mendes, Hailong Meng, Aidan Neher, Somayeh Nili, Lars Rønn Olsen, Shelby Orfield, James A. Overton, Nidhi Pai, Cokie Parker, Brian Qian, Mikkel Rasmussen, Joaquin Reyna, Eve Richardson, Sandra Safo, Josey Sorenson, Aparna Srinivasan, Nicola Thrupp, Rashmi Tippalagama, Raphael Trevizani, Steffen Ventz, Jiuzhou Wang, Cheng-Chang Wu, Ferhat Ay, Barry Grant, Steven H Kleinstein, Bjoern Peters.

Summary of feedback received after the conclusion of the challenge:

After completing the invited participant challenge, we asked participants to provide their feedback on the challenge. We sent participants a poll with questions regarding the resources, the difficulty level of the prediction tasks, future participation, and suggestions to help. The results of the feedback poll indicated that the most useful resource to participants was direct communication with the CMI-PB Consortium members (ie. emailing) and the least useful resource was the three Zoom Informational Sessions, which were held to demonstrate the submission process and the website live, as well as to encourage participants to ask questions to the CMI-PB Team in real-time.

When asked about the difficulty of the prediction tasks, with 1 being very difficult and 6 being very simple, the average response was 3.22. This showed us that the prediction tasks were evenly balanced and they were not too simple, yet not too challenging. When asked if they would participate in a similar challenge, the average response was 5.55, with 1 being very unlikely and 6 being very likely. The participants were also asked about their overall satisfaction with the challenge, and we received an average score of 5.22, with 1 being very dissatisfied and 6 being very satisfied. Overall, the feedback poll indicated that participants were satisfied with the overall experience of the prediction challenge and are looking forward to the 3rd (public) challenge.

This invited CMI-PB challenge has been designed to address some of the shortcomings identified during the first challenge. Based on the second challenge, we expect to make additional adjustments to help ensure success in the initial public challenge. This iterative process aims to provide contestants with a rich user experience, allowing for smoother data access and a much less tedious prediction submission process.

Supplementary Tables

Table A. The characteristics of all 21 subjects in the challenge dataset.

Subject ID	Age	Biological Sex at Birth	Vaccine Priming Status
97	35	Male	wP
98	28	Female	wP
99	22	Female	aP
100	20	Female	aP
101	18	Male	aP
102	18	Male	aP
103	27	Female	wP
104	32	Female	wP
105	27	Female	wP
106	25	Female	aP
107	23	Female	aP
108	26	Female	wP
109	32	Female	wP
110	24	Female	aP
111	25	Male	wP
112	25	Male	aP
114	31	Male	wP
115	19	Female	aP
116	21	Male	aP
117	27	Female	aP
118	23	Male	aP

Table B. Antibody information.

Target	Conjugate	Host	Target	Clone	Catalog	Vendor	Dilution
CD45	BUV395	Mouse	Human	HI30	563792	BD	1/500
CD8	BUV496	Mouse	Human	RPA-T8	612942	BD	1/500
CD20	BUV563	Mouse	Human	2H7	748456	BD	1/200
CD16	BV510	Mouse	Human	3G8	612786	BD	1/100
CD3	BUV805	Mouse	Human	UCHT1	612895	BD	1/200
CD14	BV480	Mouse	Human	M5E2	746304	BD	1/100
CD45RA	BV570	Mouse	Human	HI100	304132	Biolegend	1/200
CD19	BV605	Mouse	Human	HIB19	302244	Biolegend	1/200
IgD	PE-CF594	Mouse	Human	IA6-2	747484	BD	1/200
CD11c	BV785	Mouse	Human	3.9	301644	Biolegend	1/50
CCR7	FITC	Mouse	Human	G043H7	353216	Biolegend	1/66
CD123	PE-Cy7	Mouse	Human	6H6	306016	Biolegend	1/100
CD38	PerCP-Cy5.5	Mouse	Human	HIT2	562288	BD	1/50
HLA-DR	AF700	Mouse	Human	L243	307616	Biolegend	1/50
CD56	APC	Mouse	Human	5.1H11	362504	Biolegend	1/100
CD4	APC-eF780	Mouse	Human	RPA-T4	47-0049-42	LIFE TECH	1/200, 1/50
CD71	PE-Cy5	Mouse	Human	M-A712	551143	BD	1/50
CD66b	BV421	Mouse	Human	G10F5	562940	BD	1/100
CD1c	BV650	Mouse	Human	1.161	331542	Biolegend	1/200
CD141	PE	Mouse	Human	M80	47-0049-42	LIFE TECH	1/200

Supplementary Figures

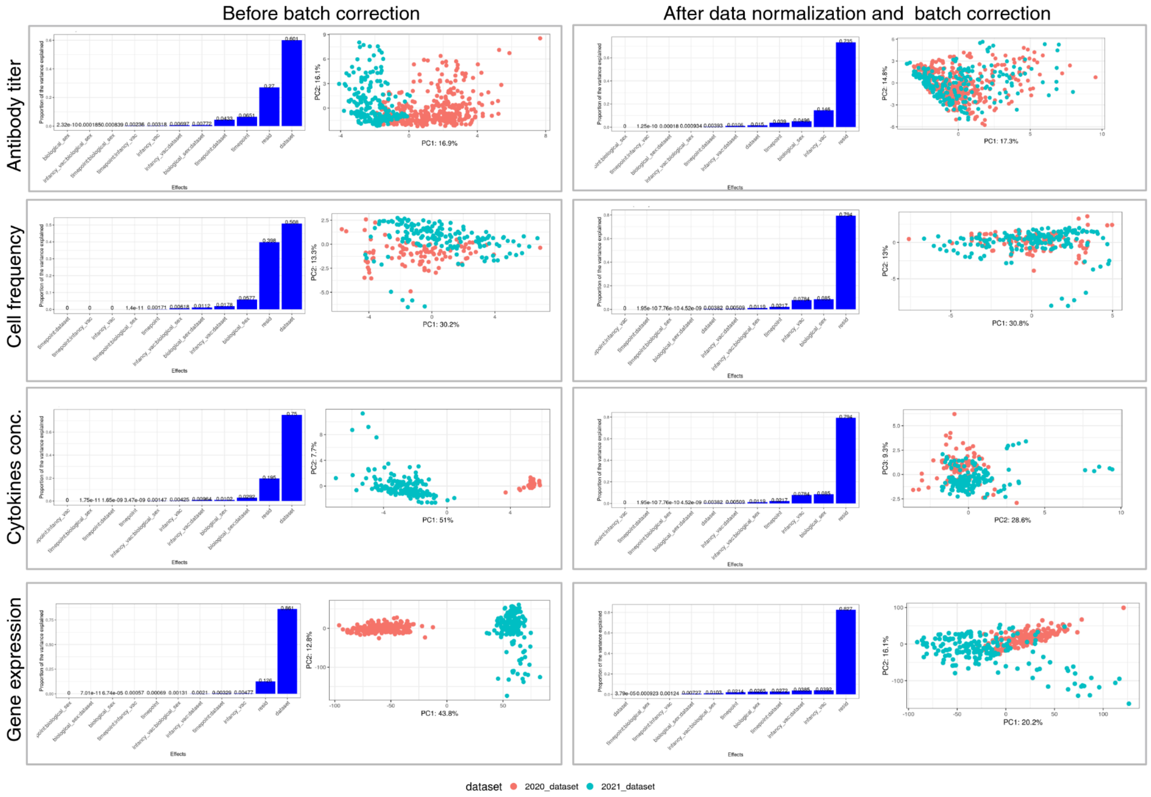

Fig A: Plot of assay data before and after normalization and batch effect correction. For each assay, the plots on the left represent data before batch correction, while the plots on the right represent data after normalization and batch correction.

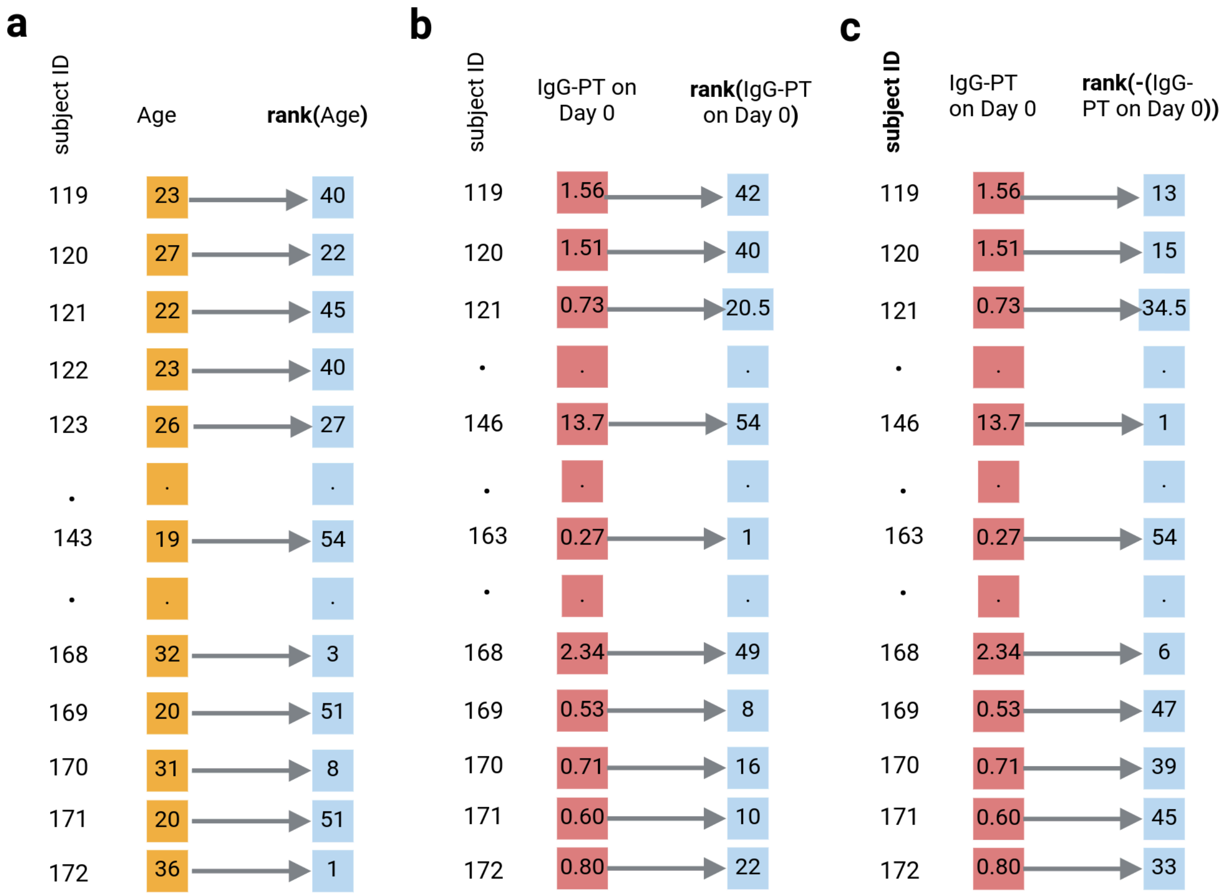

Fig B: Schematic representation of control model construction. (a) Control model using subject age: Raw age values (orange) are ranked (blue) to create a ranked list based on age. (b) The control model uses IgG-PT levels on Day 0 for Task 1.1: Raw IgG-PT values (red) are ranked (blue) to represent the relative position of subjects based on their pre-vaccination IgG-PT levels. Similarly, monocyte frequency on Day 1 was used to construct the control model for Task 2.1, and *CCL3* levels on Day 3 were used to construct the control model for Task 3.1. (c) Control model using the inverse of IgG-PT levels on Day 0 for Task 1.2: Raw IgG-PT values (red) are converted into negative values, then ranked (blue), emphasizing individuals with lower IgG-PT levels. Similarly, the negative values of monocyte frequency on Day 1 were used to construct the control model for Task 2.2, and the negative values of *CCL3* levels on Day 3 were used to construct the control model for Task 3.2.

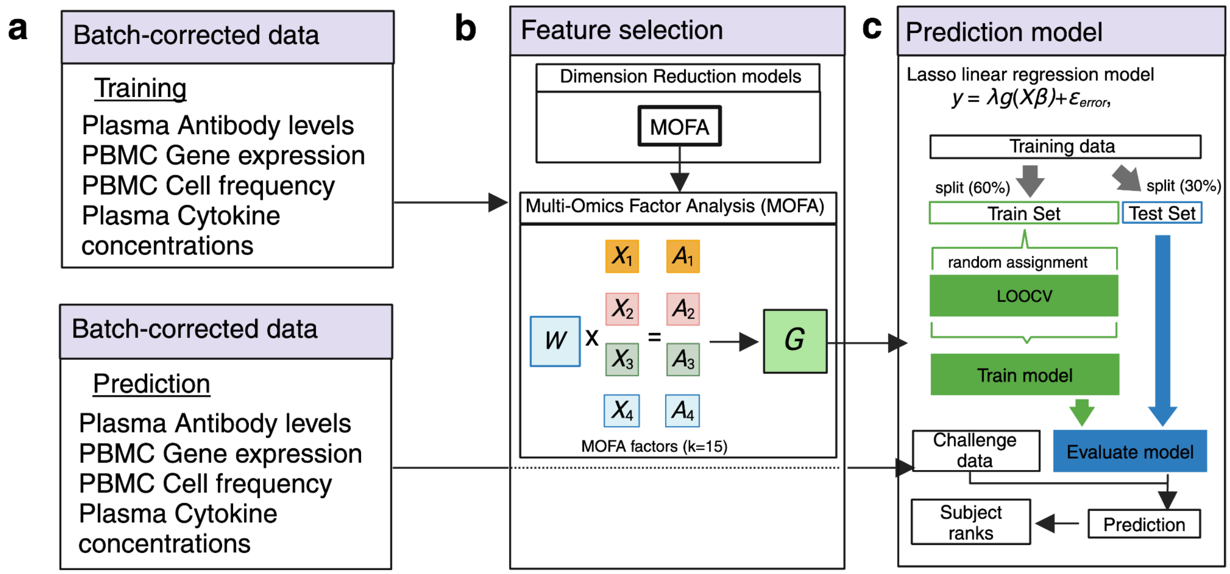

Fig C: The method implemented by Dr. Thrupp's team (user48_1). (a) The analysis pipeline begins with batch-corrected data for both training and prediction phases. The training data includes all four assay data provided, i.e., Plasma Antibody levels, PBMC Gene expression, PBMC Cell frequency, and Plasma Cytokine concentrations. Similarly, prediction data is derived from the same types of measurements. b) Multi-omics factor Analysis (MOFA) is employed to integrate multi-omics data through dimensionality reduction. The input data (X1, X2, X3, X4) represent different omics datasets (e.g., gene expression, cell frequency, cytokine levels, and antibody levels), each associated with a corresponding matrix (A1, A2, A3, A4). MOFA outputs a set of factors (G), which are used for subsequent prediction modeling. (c)

Lasso regression is used to predict the output using the selected features. The dataset is split into training (60%) and test (30%) sets. The training data undergoes leave-one-out cross-validation (LOOCV) for model training, followed by prediction on the test set. The model's performance is evaluated on the challenge baseline data, and subjects are ranked based on the challenge data outcomes submitted on the submission portal for evaluation.

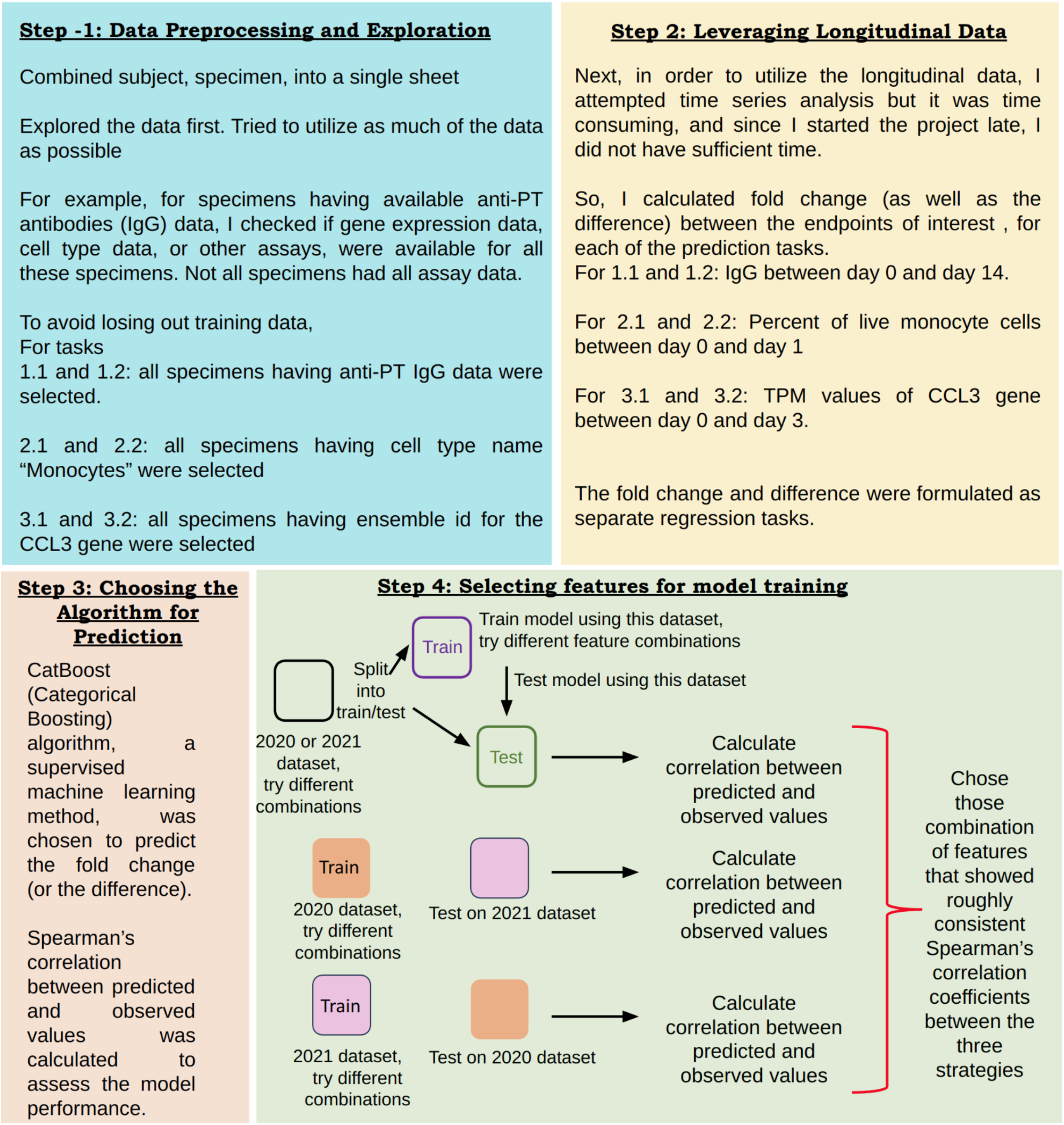

Fig D: The method implemented by Dr. Mahita's team (user54). Step 1: Data preprocessing and exploration were performed by combining subject and specimen metadata and selecting specific assays. Step 2: Leveraging longitudinal data by calculating fold change between key time points for each task. Step 3: Categorical Boosting (CatBoost) algorithm was chosen for predicting fold change or differences, with Spearman's correlation used for model evaluation. Step 4: Feature selection through model training and testing across cohort-specific datasets, with consistent correlation coefficients guiding the final feature combination.

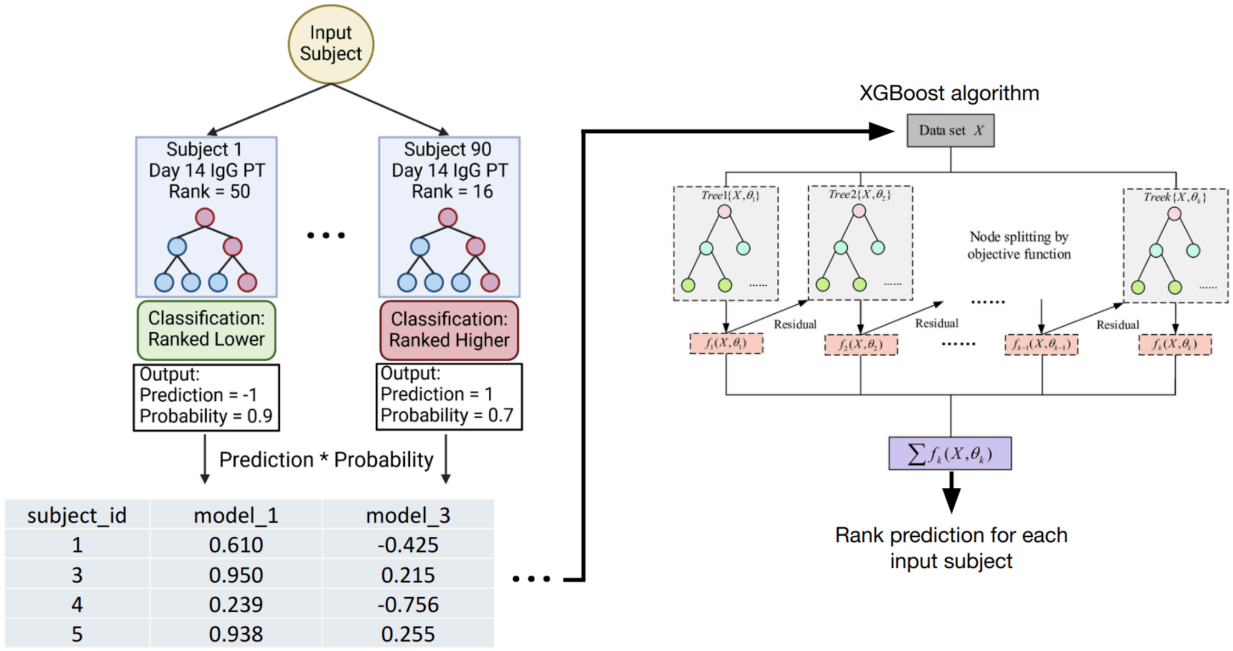

Fig E: The method implemented by Dr. Gibson's team (user51). The XGBoost algorithm workflow was developed to rank individual input subjects based on Day 14 IgG PT levels. The analysis includes 20 features from cell frequency assay, covering Monocytes (f = 4), T cell subsets (f = 12), B cells (f = 1), and Innate immune cells (f = 3); 30 Olink cytokine features such as CCL4, IL-18, and CXCL11; and 32 antibody features, including total IgG, IgG1-4, and the sum of IgG for PT, TT, PRN, FHA, DT, and OVA. (a) Input subjects (e.g., Subject 1 and Subject 90) are evaluated using a decision tree model. Each subject is assigned a classification, such as "Ranked Lower" or "Ranked Higher," based on their IgG PT level. The classification output is combined with a probability score (e.g., Subject 1: Prediction = -1, Probability = 0.9; Subject 90: Prediction = 1, Probability = 0.7), and the final prediction is calculated as the product of the prediction and probability. (b) The XGBoost algorithm processes a dataset of model summaries shown in Panel C through a series of decision trees. The algorithm iteratively refines predictions by calculating residuals after each tree and performing node splitting based on an objective function, improving accuracy over multiple trees (Tree1, Tree2, ..., Treek). The final rank prediction for each subject is determined by summing the outputs of all trees. (c) A table displays the predicted values for various subjects (subject_id) across different models (e.g., model_1, model_3), highlighting the individualized outcomes of the prediction process.

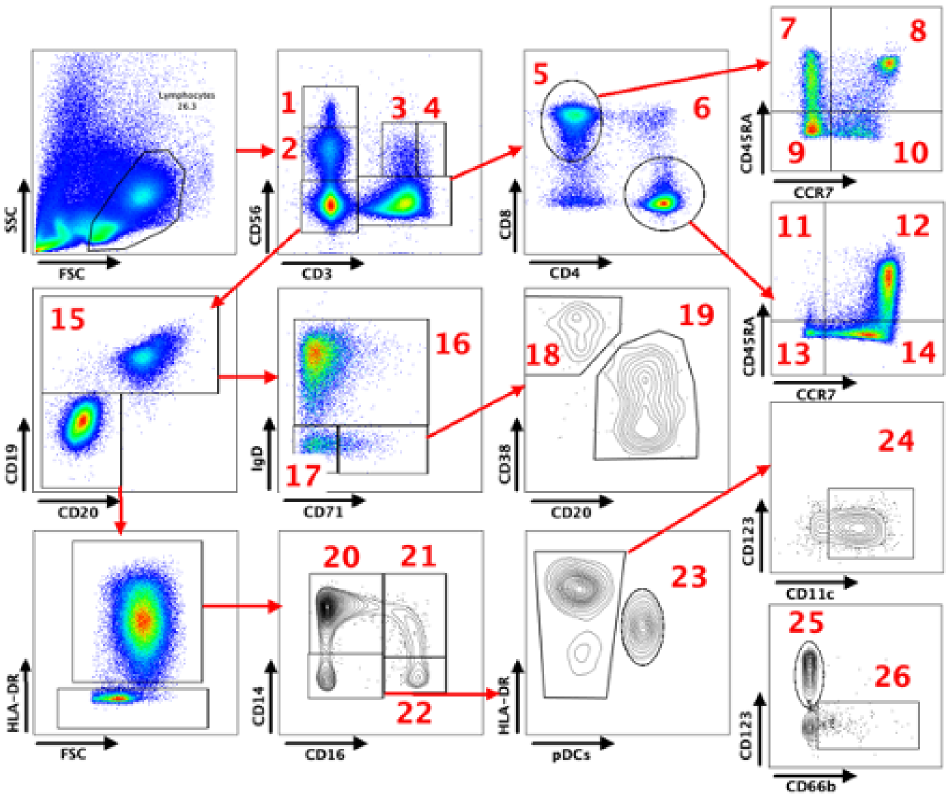

#	Cell population
1	$CD56^{high}$ NK cells
2	$CD56^{+}$ NK cells
3	$CD56^{+}$ T cells
4	$CD56^{+}$ $CD3^{high}$ T cells
5	CD8 T cells
6	CD4 T cells
7	CD8 TEMRA
8	CD8 Naïve
9	CD8 TEM
10	CD8 TCM
11	CD4 TEMRA
12	CD4 Naïve
13	CD4 TEM
14	CD4 TCM
15	B cells
16	Naïve B cells
17	Memory B cells
18	Plasmablasts
19	Activated B cells
20	Classical Monocytes
21	Intermediate Monocytes
22	Non-classical Monocytes
23	pDCs
24	Conventional DCs
25	Basophils
26	Activated granulocytes

Fig F. Gating strategy for PBMC cell frequencies (FACS).
