# Supplementary material for: Putting computational models of immunity to the test—An invited challenge to predict B.pertussis vaccination responses
Source: PLoS Comput Biol. 2025 Mar 31;21(3):e1012927. doi: 10.1371/journal.pcbi.1012927 (PMC11978014; doi:10.1371/journal.pcbi.1012927)
Supplement: Attachment — Submitted filename: PLoSCB_RebuttalDraftFeb5.pdf [file pcbi.1012927.s004.pdf]

Reviewers' comments:

Reviewer #1: In this article, the authors established a community resource to challenge computational models that predict the outcome of booster B.pertussis vaccination from a select group of scientists, evaluate and compare those models in their performance in predicting B. pertussis booster responses. They looked for the features of successful models and identified objects based on the level of difficulty in being predicted. The goal, method and results are well stated and clear. There are a few things in the paper that need to be clarified, for this reason I would suggest this paper to undertake some minor revision.

1. In this ‘invited challenge’, what is the criteria in selecting the scientists and their modeling work?

Our response: We apologize for not clearly explaining this in our previous draft. We first consulted CMI-PB investigators for their recommendations of scientists with demonstrated expertise in computational modeling, bioinformatics, or immunology. We then reviewed journal articles from PubMed and Google Scholar that either contained keywords or were cited by the originally identified authors. We then extended personalized invitations to them to participate in the CMI-PB Challenge. We have clarified this in the **Results** section (pages 3-4, Lines 119-124).

2. Are all main types of models of predicting immune response covered in this study?

Our response: When compiling the list of potential participants, our goal was to ensure a diverse representation of modeling approaches, but as stated above, it originally relied on the expertise of CMI-PB investigators. The final set of models submitted by the participating scientists encompasses classification models, linear and non-linear regression models, single- and multi-omics data integration models, and other methodologies such as gene signature analysis and module scoring. We believe this represents a substantial portion of predictive modeling strategies in the field; however, we acknowledge that some model types may not be represented. This limitation has been noted in the **Limitations of the Study** section of the revised manuscript (Page 15), and will be addressed by the next challenge, which will be open to all.

3. The tasks to be evaluated consisted of predicting plasma IgG levels against the pertussis toxin (PT) on day 14 post-booster vaccination and the fold change of the plasma IgG levels against PT between day 14 post-booster vaccination and baseline, predicting the overall frequency of monocytes among PBMCs on day 1 post-booster vaccination and the corresponding fold change and the CCL3 gene expression on day 3 post-booster vaccination and the corresponding fold change values compared to baseline. Is there a reason that those prediction are tested at those specific days (14, 3, 1 post booster vaccine)?

Our response: We selected biological readouts known to be changed by booster vaccination under the premise that they are likely to capture meaningful heterogeneity across study participants based on our previous work (PMID:33690224).

- **Tasks 1.1 and 1.2 (IgG_PT on Day 14)** were chosen for plasma IgG levels, as they typically mark the peak antibody response period around two weeks post-booster vaccination.
- **Task 2.1 and 2.2 (Monocytes on day 1):** We have shown that the percentage of monocytes was significantly elevated on day 1 post-booster vaccination compared to baseline (i.e., before booster vaccination), highlighting the role of monocytes in Tdap vaccine response.
- **Tasks 3.1 and 3.2 (*CCL3* on day 3):** Our previous finding was that a subset of aP-primed individuals showed an increased expression of proinflammatory genes, including *CCL3*, on day 3 post-booster vaccination.

For clarity, these rationales have been added to the **Methods** section (page 15, lines 582-593) of the revised manuscript. We hope this addresses the reviewers' concerns.

4. Are the evaluation and comparison conducted on the list of ranks for the 21 subjects for each one of the 6 tasks? Is there any evaluation on the values of plasma IgG levels, overall frequency of monocytes among PBMCs or the CCL3 gene expression?

Our response: The challenge evaluation and model comparisons were exclusively based on the ranked predictions for the 21 subjects across the 6 tasks. Contestants were instructed to calculate the ranks of the predicted values generated by their models and submit these ranks for evaluation. We used the Spearman correlation coefficient as the evaluation metric, as it is non-parametric and well-suited for data with non-normal distributions, which were characteristic of the analyte measurement outputs from the different experimental assays utilized in the study.

The evaluation process consisted of two steps:

- Step 1 (Calculate correlation coefficient): Spearman correlation coefficients were calculated between the observed values and the ranks of the predicted values submitted by participants for each plasma IgG level, monocyte frequencies among PBMCs, and *CCL3* gene expression tasks.
- Step 2 (Performance Scoring): Based on performance, points were assigned to each task, allowing for the ranking of all submissions and the identification of winners.

The prediction tasks in our first challenge involved predicting the rank of individuals in specific immune response readouts, ranging from high to low, after *B. pertussis* booster vaccination based on their pre-vaccination status. By exclusively analyzing relative rankings, the evaluation ensured robust comparisons through ranked predictions without reliance on raw data values. For clarity, we have revised the methods (Page 16; lines: 606-609) and results (Page 7; lines: 274-277) sections to include these details.

Reviewer #2: Shinde et al. provide a nice overview of the third competition in the Computational Models of Immunity to Pertussis Booster (CMI-PB) challenge series. In their paper, the authors discuss the outcomes of a model development/prediction task undertaken by 20 teams, that also included two "control" models and 22 literature based models. The goal of this competition is noteworthy and raises important questions about the applicability of models developed to

understand pertussis vaccination. I have some suggestions for additions that I encourage the authors to consider:

1. Clarifying what "model" means here would be useful for a broader audience. The winning team used machine learning, regression models. As a mechanistic modeller, I was a bit unsure throughout the text what the structure of the models were and were they statistical vs mathematical vs computational etc. The authors do provide a list of the top 5 + "other" model types, but some more discussion would help clarify.

Our response: We thank the reviewer for suggestions regarding defining the term "model" for a broader audience, which we indeed used as a 'catch-all' for different approaches. To clarify this, we have added a detailed explanation in the manuscript to describe what we mean by "model" in the context of this prediction challenge (pages 6-7, lines 228-231; 236-249), and that it does not imply that these were mechanistic models.

The contestants' submitted models encompass multiple steps, and they used multiple tools to draft their models. For example, the winning model first performed data imputation and batch effect correction, followed by MCIA for multiomics dimensionality reduction. The results of this step were then used to build a prediction model employing LASSO regression with leave-one-out cross-validation.

We have also expanded the description of the top-performing models (page 8-9, lines: 303-345; page 9,10,11, across lines: 373-419). We believe these additions provide a clearer understanding for readers from diverse scientific backgrounds.

2. Similarly, the control models were unclear to me. Control model 1 used age to rank (?) participants it seems. Is it a regression model with age as the regressor? Maybe a figure panel or equations laying out both control models would be useful.

Our response: This is likely related to the previous reviewer's comment and our broad use of the term 'model'. To clarify, both control 'models' were simply using a single variable, so the 'age model' simply ranked study subjects based on their chronological ages. To improve clarity, we have included Supplementary Figure S6 in the revised manuscript outlining the methodology for both control models and how the rankings were calculated and referred to in the Results section (Page 6, lines 210-213). We hope this addresses your concern and provides a clearer understanding of the control models.

3. I was interested in the commonalities between the highest ranked models. Were there any biological features of well-ranked (say top 5) conserved across models. If so, what do they help us learn about pertussis booster vaccination. Some overview is given in the discussion. However, given the unique nature of this challenge and the data the team has assembled, it would be nice to also provide key biological take-away messages.

Our response: We thank the reviewer for the insightful comment regarding the commonalities among the highest-ranked models and their biological implications. While there was no clear consensus on specific methodologies at the modeling level among the top-performing

submissions, we observed that these models have overall common features: handling nonlinearities, reducing large feature sets to more manageable subsets, and utilizing advanced data preprocessing techniques. After the challenge, we requested model summaries from all participating teams. While most contestants provided details on the data they used, processing steps, imputation methods, and feature selection approaches, fewer teams submitted information on the top-performing features. As a result, we focused on the winning team models to identify the key contributors. Using this information, we expanded the description of the biological understanding of each task on pages 8-9 (lines 303-345) and also revised Table 2 with these details. We have taken note of this point for our future public challenges. We will proactively request contestants to submit information on the top-performing features to better capture and highlight the most relevant contributors for top models.

4. For the normalization, was there any removal of background measures when calculating the median baseline concentration?

Our response: Thank you for your question. The normalization process involved three steps:

1. All zero values were replaced with the limit of detection (LOD) to account for background noise. The LOD was calculated as half the value of the first non-zero measurement for each feature.
2. The median baseline concentration was first calculated using the adjusted dataset. This value was then used to normalize all data points by dividing each value by the median baseline concentration.

The revised manuscript clarifies these details in the **Methods** section (page 15, lines 563-566). We hope this addresses your concern.

I also had a few minor comments:

1. On page 4, it may be clearer to move the sentence "The repeat pre-vaccination samples were intended to give a stable estimate of baseline and variability." to after the discussion of the measurement taken from each specimen (otherwise one wonders "baseline and variability of what?").

Our response: We thank the reviewer for the suggestions in the manuscript. We moved the mentioned statement after discussing the measurements taken.

2. Also on page 4, "such as IL-6 cytokine in the plasma cytokine" could simply be "such as IL-6 in the plasma..."

Our response: We revised the text.

3. I think there is a missing reference on page 5 ("[38490204]").

Our response: We fixed the missing reference on the page 5.

4. Page 11: "The detailed description is provided here11." Maybe "is provided in da Silva Antunes et al.11" is better?

Our response: As the reviewer kindly suggested, we revised the text in two places (Page:, lines:).

Reviewer #3: The manuscript presents an important contribution to the field of systems vaccinology. It effectively demonstrates the value of community-driven benchmarking in the computational modeling of vaccine responses and provides a robust framework for evaluating predictive models. The study is well-designed, and its findings offer meaningful insights into the methodologies that improve predictive accuracy and reliability in this context.
The manuscript is well-written, and the dataset and methodology are clearly described. The open science approach, with datasets and code made publicly available, aligns with best practices for transparency and reproducibility in research. The establishment of a community resource and the invitation for future challenges further enhance the relevance and impact of the study.

Our response: We thank the Reviewer for their positive feedback and recognition of our contribution to systems vaccinology. We greatly appreciate the reviewers' acknowledgment of the community resource and future challenges, and we remain committed to fostering collaboration and advancing vaccine research. We thank the reviewer for their thoughtful review and suggestions, which helped us strengthen the manuscript.

There are only a few minor points that could be addressed to strengthen the clarity and accessibility of the manuscript:
1. Tables are somewhat dry (i.e. “boring”) in presentation. It would be helpful to visualize some of these results for better engagement and understanding.

Our response: We thank the reviewer for suggesting modifications to the table structures in the manuscript. In response, we have revised the tables to include additional information. Table 2 now highlights how many models demonstrated significant Spearman correlation coefficients for each task. Similarly, we have updated Table 3 to include model performance rankings. We believe these revisions make Table 2 and Table 3 more results-oriented and engaging.

2. The manuscript acknowledges the limited size of the challenge dataset, but including a brief discussion on how this limitation could be addressed in future challenges would provide a more balanced perspective. Additionally, it would be beneficial to comment on the dataset specificity of the models and their potential generalizability.

Our response: We thank the reviewer's insightful suggestion. We have included a brief discussion of how we planned to address the limitations of the smaller challenge dataset in future challenges, such as expanding the dataset and incorporating more assays (Page: 17, lines: 632-640).

3. Adding a few sentences to highlight how the findings, particularly from a biological perspective, align with or diverge from previous systems vaccinology studies would enhance the contextual significance of the work.

Our response: We thank the reviewer's suggestion to provide a broader biological context for our findings. In response, we have added sentences to discuss how our results align with or diverge from previous systems vaccinology studies (page: 12, lines: 475-473). Specifically, we highlight consistencies in key immune signatures, such as cytokine (IL-6, IFN-γ) profiles that have been previously linked to vaccine efficacy. Additionally, we address unique findings, including the importance of purpose-specific models to better capture the biological signals within the dataset. These comparisons emphasize the biological relevance of our findings and offer valuable context for interpreting them within the broader framework of systems vaccinology.

4. While the conclusion is robust, it could be strengthened by outlining how the findings might influence vaccine development or immunological research.

Our response: We appreciate the reviewer's suggestion. We have expanded the conclusion to highlight how our results could influence vaccine development and immunological research (page 13, lines 500-508).
